# Determination of high‐sensitivity cardiac troponin T upper reference limits under the improved selection criteria in a Chinese population

**DOI:** 10.1002/jcla.23007

**Published:** 2019-08-16

**Authors:** Xin Zhang, Xiaoxu Han, Min Zhao, Runqing Mu, Shuo Wang, Ke Yun, Hong Shang

**Affiliations:** ^1^ Department of Laboratory Medicine The First Affiliated Hospital of China Medical University Shenyang China; ^2^ National Clinical Research Center for Laboratory Medicine The First Affiliated Hospital of China Medical University Shenyang China

**Keywords:** 99th percentile, high‐sensitivity cardiac troponin T, reference population, selection criteria

## Abstract

**Background:**

There is no common consensus on how to define the reference population for determination of high‐sensitivity cardiac troponin (hs‐cTn) upper reference limit (URL). This study aimed to establish 99th percentile URLs of hs‐cTnT under both 2018 AACC/IFCC criteria and improved selection criteria for further judging whether two URLs are different.

**Methods:**

Applying the stratified cluster sampling protocol, this study took 1848 apparently healthy subjects in communities of Shenyang China as the screening objects. We first followed 2018 AACC/IFCC criteria using surrogate biomarker for diabetes, myocardial dysfunction, renal dysfunction, and electrocardiogram. Then, we followed improved selection criteria to exclude hypertension, overweight and obesity, and dyslipidemia by physical examination and laboratory screening. Accordingly, 99th percentile URLs of hs‐cTnT were established.

**Results:**

If the 2018 AACC/IFCC criteria were applied, 99th percentile URLs (90% confidence interval) of hs‐cTnT male, female, and total were 19 (17‐20) ng/L, 16 (15‐17) ng/L, and 18 (16‐19) ng/L, respectively. If added a single supplementary selection criteria, 99th percentile URLs of hs‐cTnT total reduced to 16 ng/L, 17 ng/L, and 16 ng/L, respectively. If the improved selection criteria were applied, 99th percentile URLs (90% confidence interval) of hs‐cTnT male, female, and total were 18 (14‐24) ng/L, 13 (11‐16) ng/L, and 16 (13‐17) ng/L, respectively. The 99th percentile URLs of hs‐cTnT male were higher than those of female in every age group.

**Conclusions:**

Improved selection criteria through questionnaire survey, physical examination, and laboratory screening to further exclude hypertension, overweight and obesity, and dyslipidemia can avoid overestimation of the 99th percentile URL of hs‐cTnT.

## 1 INTRODUCTION

In light of the fourth universal definition of myocardial infarction (MI) announced in 2018 and 2015 European Society of Cardiology (ESC) guidelines for the management of acute coronary syndromes (ACS) in patients presenting without persistent ST‐segment elevation, cardiac troponin (cTn), especially high‐sensitivity cardiac troponin (hs‐cTn), is the preferred biomarker for the diagnosis of MI.^1^, ^2^

It is important to establish 99th percentile upper reference limit (URL) of cTn, which is widely acknowledged in the universal definitions of myocardial injury and different types of MI.^1^ For myocardial injury, cTn should elevate at least one value above 99th percentile URL. For types 1, 2 and 3 MI, cTn should rise and/or fall at least one value above 99th percentile URL. For type 4a MI (percutaneous coronary intervention–related MI) and type 5 MI (coronary artery bypass grafting–related MI), cTn should elevate more than 5 times and 10 times of 99th percentile URL, respectively.

However, up to now there is no consensus on how to define reference population. The CLSI document Reference Intervals in the Clinical Laboratory (EP28‐A3c)^3^ recommended a questionnaire‐based approach for determining laboratory test reference intervals. The present 99th percentile URL of hs‐cTnT (14 ng/L) was established based on 616 “apparently healthy” volunteers and blood donors, but little information was reported about population selection,^4^ and then, it was confirmed by Saenger et al^5^ based on 533 individuals with a standardized questionnaire from the United States and Europe. But Collinson et al,^6^ Koerbin et al,^7^ and McKie et al^8^ compared questionnaire‐screened strategy and other supplementary strategies and then found simply using self‐reporting of health was insufficient in defining the reference population of hs‐cTn. Prof. Yader Sandoval and Prof. Fred S. Apple's recommendations for defining a normal reference population for the determination of 99th percentile of hs‐cTn should include clinical history for known cardiovascular disease and medication usage, surrogate biomarker for diabetes, surrogate biomarker for myocardial dysfunction, surrogate biomarker for renal dysfunction, inclusion of an imaging modality if financially feasible, and diverse population of sufficient sample size (minimum 300 men and 300 women),^9^ which also admitted by International Federation of Clinical Chemistry Task Force on Clinical Applications of Cardiac Biomarkers (IFCC TF‐CB).^10^ In the recent published clinical laboratory practice recommendations for the use of cTn in ACS: expert opinion from the academy of the American Association for Clinical Chemistry and IFCC TF‐CB (we call it 2018 AACC/IFCC criteria for short), Wu et al^11^ recommended that imaging modality was essential although financial burden for clinical laboratories and in vitro diagnostic companies. In our opinion although not recommended which should be chosen MRI, echocardiogram, or electrocardiogram (ECG) in a broad sense, the main purpose was to exclude potential patients not only through questionnaire.^12^

The chief objective of screening was to exclude cardiovascular disease and related disease, especially the ones which might influence hs‐cTn values. There was still a possibility that 2018 AACC/IFCC criteria, through questionnaires, surrogate biomarkers, and imaging, might omit these important cardiovascular risk factors,^13^ such as hypertension, overweight and obesity, and dyslipidemia. Without blood pressure measurements, hypertension was likely to be missed only through questionnaire. For example, in China, the prevalence and awareness of hypertension were 37.2% and 36.0%, respectively,^14^ and the awareness rate still exited in the United States^15^ and other countries.^16^, ^17^, ^18^ For obesity and overweight, it was reported that it affected about two‐thirds of the Americans,^19^ and previous study showed that hs‐cTnI was dependent on BMI in their multiple linear regression model.^20^ For blood lipid, Lippi et al^21^ found that high‐density lipoprotein cholesterol (HDL‐C) inversely and independently predicted hs‐cTnT. In addition, other researchers also proved positive associations between hs‐cTnT and atherogenic lipid markers, such as total cholesterol (TC), triglyceride (TG), and low‐density lipoprotein cholesterol (LDL‐C).^22^

To summarize, the objective of our study was to establish 99th percentile URLs of hs‐cTnT (Roche Diagnostics) under both 2018 AACC/IFCC criteria and improved selection criteria through questionnaire survey, physical examination, and laboratory screening to further exclude hypertension, overweight and obesity, and dyslipidemia in a Chinese population, further judging whether two 99th percentile URLs of hs‐cTnT established are different.

## 2 MATERIALS AND METHODS

### 2.1 Study population and inclusion/exclusion criteria

**Figure 1:**
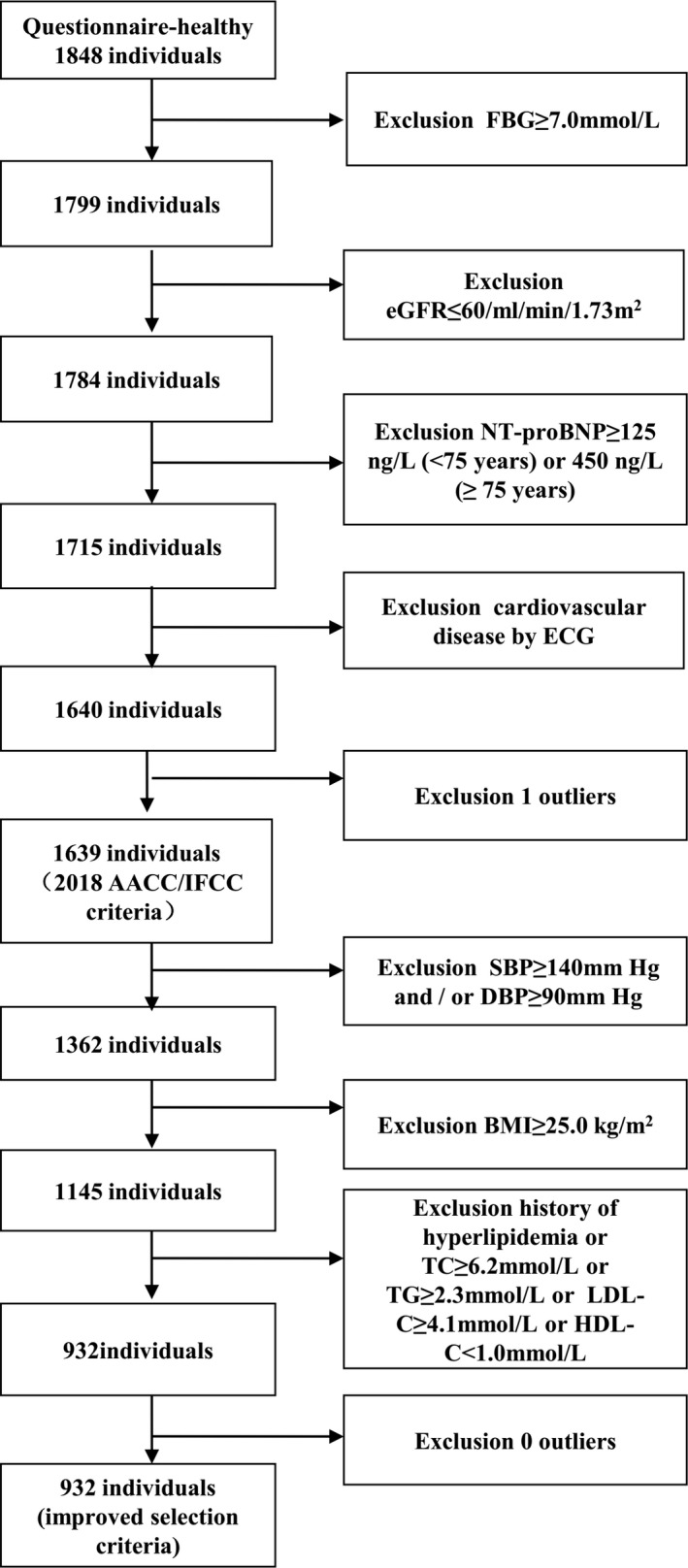
Procedures for selection of the individuals on establishing 99th percentile URL of hs‐cTnT

This study was approved by the ethics committee of the First Hospital of China Medical University. All individuals signed informed consent before the study. From January to July in 2014, applying the stratified cluster sampling protocol, this study took 1848 apparently healthy subjects through questionnaire (with no known cardiovascular disease or related disease and medication history, such as coronary heart disease (angina, MI), heart failure, arrhythmia, hypertension, stroke (cerebral hemorrhage, cerebral infarction, subarachnoid hemorrhage), diabetes) in communities of Shenyang China as the screening objects. Blood donation, blood transfusion, pregnancy, and individuals younger than 18 years of age were excluded. Fasting blood glucose (FBG) ≥ 7.0 mmol/L was used to represent surrogate biomarker for diabetes. Estimated glomerular filtration rate (eGFR) ≤ 60/mL/min/1.73 m^2^ was used to represent surrogate biomarker for renal dysfunction. Aminoterminal pro–B‐type natriuretic peptide (NT‐proBNP) ≥ 125 ng/L (<75 years) or 450 ng/L (≥75 years) was used to represent surrogate biomarker for myocardial dysfunction. Electrocardiogram was used to exclude ventricular hypertrophy, atrial hypertrophy, WPW syndrome, MI, atrial fibrillation, atrial flutter, supraventricular tachycardia, ventricular tachycardia, premature beat, and atrioventricular block. According to this procedure after exclusion outliers, 1639 individuals were enrolled in our study under 2018 AACC/IFCC criteria. Furthermore, we applied stricter exclusion criteria (such as hypertension (systolic blood pressure (SBP) ≥ 140 mm Hg and/or diastolic blood pressure (DBP) ≥ 90 mm Hg), dyslipidemia (with history of dyslipidemia or TC ≥ 6.2 mmol/L or TG ≥ 2.3 mmol/L or LDL‐C ≥ 4.1 mmol/L or HDL‐C < 1.0 mmol/L),^23^ overweight and obesity (BMI ≥ 25.0 kg/m^2^)^24^). According to this procedure, 932 individuals enrolled in our study under improved selection criteria. Figure 1 summarized specific screening process and the exact exclusion numbers during each step.

### 2.2 Laboratory analysis

All subjects maintained normal working and dietary habits 1 week before blood collection, avoided strenuous exercise and physical labor, avoided heavy drinking, prohibited drinking 1 day before blood collection, and avoided overeating at night and high‐fat and high‐protein diet; fasting time was 8 hours to 14 hours after dinner (including drinks, milk, fruits, etc), and water consumption was not more than 200 mL before blood collection. Smoking and vigorous exercise are prohibited for one hour. Blood samples were collected from the elbow vein and allocated to 5 mL SST tube with gel (Becton Dickinson, USA). All blood samples were centrifuged with 1200 *g* for 10 minutes within 2 hours after collection, and serum was analyzed within 2 hours after separation.

We measured concentrations of FBG, TG, TC, HDL‐C, LDL‐C, and creatinine (Cr) by using Roche Modular automatic biochemical analyzer (Roche Diagnostics). We measured concentrations of NT‐proBNP and hs‐cTnT on a Cobas E170 Analyzer (Roche Diagnostics).

### 2.3 Statistical analysis

The statistical analysis of this study was performed using SPSS 22.0. The Mann‐Whitney test was used for comparison between two subgroups. Outliers were identified in the total population and in male and female separately using the methods of Dixon^3^ and Reed.^25^ After excluding the outliers, the 99th percentile URLs and 90% confidence interval were calculated using nonparametric method.^26^

## 3 RESULTS

### 3.1 Study population

Characteristics of the study population are presented in Table 1. Under the improved selection criteria, a total of 932 individuals were enrolled. Han nationality occupied 89.7% and 81.1% individuals from urban area. A total of 345 (37.0%) were male gender and 587 (63.0%) were female gender. Under the 2018 AACC/IFCC criteria, a total of 1639 individuals were enrolled. Han nationality occupied 90.1% and 81.9% individuals from urban area. A total of 791 (48.3%) were male gender and 848 (51.7%) were female gender. Study participants were divided into five age groups including 18‐29 years old, 30‐39 years old, 40‐49 years old, 50‐59 years old, and ≥60 years old. There were 276 individuals, 208 individuals, 187 individuals, 160 individuals, and 101 individuals in each group under improved selection criteria. There were 371 individuals, 330 individuals, 347 individuals, 317 individuals, and 274 individuals in each group under 2018 AACC/IFCC criteria. Compared with improved selection criteria group, there were 277 (16.9%) hypertension individuals, 309 (18.9%) overweight and obesity individuals, and 422 (25.7%) dyslipidemia individuals among 1639 individuals in 2018 AACC/IFCC criteria group.

**Table 1:** Characteristics of the study population

Variable	Improved selection criteria^b^ N (%)	2018 AACC/IFCC criteria^a^ N (%)
Total	932(100)	1639(100)
Han nationality	836(89.7)	1477(90.1)
Ethnic minority	96(10.3)	162(9.9)
Urban	756(81.1)	1343(81.9)
Rural	176(18.8)	296(18.1)
Male	345(37.0)	791(48.3)
Female	587(63.0)	848(51.7)
Age group (y)		
18‐29	276(29.6)	371(22.6)
30‐39	208(22.3)	330(20.1)
40‐49	187(20.1)	347(21.2)
50‐59	160(17.2)	317(19.3)
≥60	101(10.8)	274(16.7)
Hypertension	0	277(16.9)
Overweight and obesity	0	309(18.9)
Dyslipidemia	0	422(25.7)

a Minimum 300 men and 300 women + questionnaire + surrogate biomarker for diabetes + surrogate biomarker for myocardial dysfunction + surrogate biomarker for renal dysfunction + ECG.

b Footnote a + other supplementary examinations to exclude individuals with hypertension, overweight and obesity, and dyslipidemia.

### 3.2 The 99th percentile URLs of hs‐cTnT under different selection procedures

The 99th percentile URLs of hs‐cTnT under different selection procedures are presented in Table 2. If the 2018 AACC/IFCC criteria were applied, 99th percentile URLs (90% confidence interval) of hs‐cTnT male, female, and total were 19 (17‐20) ng/L, 16 (15‐17) ng/L, and 18 (16‐19) ng/L, respectively. If added a single supplementary selection criteria, hypertension or overweight and obesity, or dyslipidemia, 99th percentile URLs of hs‐cTnT male and female remained the same; however, 99th percentile URLs of hs‐cTnT total reduced to 16 ng/L, 17 ng/L, and 16 ng/L, respectively. If the improved selection criteria were applied, 99th percentile URLs (90% confidence interval) of hs‐cTnT male, female, and total were 18 (14‐24) ng/L, 13 (11‐16) ng/L, and 16 (13‐17) ng/L, respectively.

**Table 2:** The 99th percentile URLs of hs‐cTnT under different selection procedures

Selection criteria	N	99th percentile URL (90% CI, ng/L)		
		Male	Female	Total
2018 AACC/IFCC criteria^a^	1639	19 (17‐20)	16 (15‐17)	18 (16‐19)
2018 AACC/IFCC criteria^a^ + hypertension	1362	19 (16‐21)	16 (13‐17)	16 (15‐19)
2018 AACC/IFCC criteria^a^ + overweight and obesity	1330	19 (17‐20)	16 (13‐17)	17 (16‐19)
2018 AACC/IFCC criteria^a^ + dyslipidemia	1217	19 (16‐21)	16 (13‐16)	16 (16‐18)
2018 AACC/IFCC criteria^a^ + hypertension, overweight and obesity, and dyslipidemia	932	18 (14‐24)	13 (11‐16)	16 (13‐17)

a Minimum 300 men and 300 women + questionnaire + surrogate biomarker for diabetes + surrogate biomarker for myocardial dysfunction + surrogate biomarker for renal dysfunction + ECG.

### 3.3 Distribution of hs‐cTnT values by age and gender under improved selection criteria

Distribution of hs‐cTnT values by age and gender under improved selection criteria is presented in Table 3. The 25th percentile, median, and 75th percentile of hs‐cTnT male were higher than those of female in every age group (*P* = .004 in ≥60 years old and *P* < .001 in other age groups). The 99th percentile URLs of hs‐cTnT male were higher than those of female in every age group. The 99th percentile URLs of hs‐cTnT increased with age in both genders except for 18‐29 years old and 30‐ to 39‐year‐old men. The difference in the 99th percentile URLs of hs‐cTnT between women aged 50‐59 and over 60 was very small (18 ng/L vs 19 ng/L).

**Table 3:** Distribution of hs‐cTnT values by age and gender under improved selection criteria

Age group (y)	N	hs‐cTnT male (ng/L)		hs‐cTnT female (ng/L)		*P* value
		Median (25th, 75th percentiles)	99th percentile	Median (25th, 75th percentiles)	99th percentile	
18‐29	276	6 (5, 7)	11	5 (4, 5)	7	<.001
30‐39	208	6 (5, 7)	11	4 (4, 5)	8	<.001
40‐49	187	6 (6, 7)	16	5 (4, 5)	9	<.001
50‐59	160	8 (6, 9)	19	6 (5, 7)	18	<.001
≥60	101	9 (7, 11)	28	7 (6, 9)	19	.004

### 3.4 Summary of 99th percentile URLs of hs‐cTnT among different races under 2018 AACC/IFCC criteria

Table 4 summarizes the 99th percentile URLs of hs‐cTnT among different races under 2018 AACC/IFCC criteria. Besides our study, two studies established 99th percentile URLs of hs‐cTnT under 2018 AACC/IFCC criteria. The Italian study followed 2018 AACC/IFCC criteria except for Pisa cohort (n = 182) and Bolzano cohort (n = 290) without imaging modality and then excluded hypertension and obesity whose standard was different from ours (BMI ≥ 30 kg/m^2^ vs BMI ≥ 25 kg/m^2^) but without dyslipidemia exclusion. The Dutch study followed 2018 AACC/IFCC criteria but without supplementary selection criteria. The 99th percentile URLs of hs‐cTnT varied between races under different selection criteria. The 99th percentile URL of hs‐cTnT male was higher than that of female, and the 99th percentile URL of hs‐cTnT elderly persons was higher than that of younger persons in the Italian study.

**Table 4:** Summary of 99th percentile URLs of hs‐cTnT among different races under 2018 AACC/IFCC criteria

Race	N	Supplementary selection criteria			99th percentile URL (90% CI/ 95% CI^a^, ng/L)		
		Hypertension	Overweight and obesity	Dyslipidemia	Male	Female	Total
Chinese	932	Yes	Yes	Yes	18 (14‐24)	13 (11‐16)	16 (13‐17)
Italian^b^	1047	Yes	Obesity	No	20‐64:23.2 (17.3‐34.1) ≥65:36.8 (21.7‐37.0)	20‐64:10.2 (8.5‐21.9) ≥65:28.6 (17.6‐28.6)	20‐64:19.9 (14.4‐27.5) ≥65:33.7 (21.0‐37.0)
Dutch^c^	1535	No	No	No	16 (15‐17)	12 (10‐14)	15 (13‐16)

a Chinese study uses 90% CI; Italian and Dutch study uses 95% CI.

b Franzini et al^27^; Pisa cohort (n = 182) and Bolzano cohort (n = 290) did not refer to 2018 AACC/IFCC criteria.

c Kimenai et al.^28^

## 4 DISCUSSION

Being a single‐center study, our research programs are more representative such as stratified cluster sampling protocol but not volunteers nor blood donors, which avoided selection bias. The Han nationality accounted for around 90% of the individuals enrolled in our study under two selection criteria, which accorded with the composition of the Chinese population in the sixth nationwide population census. The ethnic distribution and urban‐rural distribution under two selection criteria were basically the same, indicating that it was not the result of selective bias. Our improved selection criteria can be used by other researches.

To the best of our knowledge, we conducted the first study to determine 99th percentile URLs of hs‐cTnT under both 2018 AACC/IFCC criteria and improved selection criteria through questionnaire survey, physical examination, and laboratory screening to further exclude hypertension, overweight and obesity, and dyslipidemia, which was proven in our study that the latter criteria avoided overestimation of the former. Compared with other two studies for 99th percentile URLs of hs‐cTnT among different races under 2018 AACC/IFCC criteria,^27^, ^28^ our screening strategy was also the most comprehensive. The importance of reference population selection criteria has been emphasized in previous studies. In our study, there were still 16.9% hypertension, 18.9% overweight and obesity, and 25.7% dyslipidemia under the 2018 AACC/IFCC criteria. Furthermore, we found the 99th percentile URLs of hs‐cTnT total could be affected by a single supplementary selection criteria, hypertension, or overweight and obesity, or dyslipidemia. Compared with previous studies, in which the relationship was concluded from either indirect multiple linear regression model^20^ or patients,^21^, ^22^ our studies provided direct evidence. However, in a subcohort (n = 304) of an European study, 99th percentile URLs of hs‐cTnI total remained the same (10.8 ng/L) after exclusion participants with dyslipidemia, although 99th percentile URLs of hs‐cTnI total were different between participants with dyslipidemia and normal lipid (12.8 ng/L vs 10.8 ng/L).^29^

Gender is an important factor affecting the 99th percentile URLs; therefore, when using hs‐cTn, gender‐specific 99th percentile URLs are recommended.^10^, ^11^ Together with other two studies of 99th percentile URLs of hs‐cTnT for Italian^27^ and Dutch^28^ under 2018 AACC/IFCC criteria, we all found 99th percentile URL of hs‐cTnT male was higher than that of female. This attributed to intrinsic differences between genders that the concentration of cTn was correlated with left ventricular mass, which was higher in men than in women.^30^, ^31^, ^32^

The physiological difference between genders was one thing but whether or to what extent it would affect clinical management and improve outcomes still needs further studies. There were two studies using gender‐specific URLs of hs‐cTnT (15.5 ng/L for men and 9 ng/L for women), but reclassification has little effect on diagnosis and prognosis. Mueller‐Hennessen et al^33^ enrolled 1282 unselected suspected AMI patients at the emergency department from part of the TRAPID‐AMI study, then found that AMI rate increased 6% in entire cohort and 9.1% in ACS subcohort for women, whereas for men, rates decreased 2% in entire cohort and 2.9% in ACS subcohort. Rubini Giménez et al^34^ enrolled 2734 suspected AMI participants at the emergency department, but only two women's diagnosis changed from unstable angina to AMI and one man's diagnosis changed from AMI to unstable angina, which totally possessed 0.11% of all patients and 0.6% of patients with AMI. In the aspect of assessing acute cardiac outcome, Kavsak et al^35^ found there was no difference in the concentrations of hs‐cTnT between genders. We expected that proper gender‐specific cutoffs under uniform and improved selection criteria would be used to assess outcomes in future studies.

Considering no guidelines recommended age‐specific 99th percentile URLs till now,^11^ we did not give it but we saw the increasing trend in our study. In the past, population‐based studies showed that hs‐cTn concentrations increased with age.^36^, ^37^ Because the data were collected from unselected population, comorbidities were inevitable. Apple et al^12^ thought the increasing trend might due to comorbidity, and age‐specific 99th percentile URLs would increase complexity, which is detrimental to healthier elderly. But Franzini et al^27^ found the increasing trend still exited in highly selected healthy population. We confirmed it in our population, in which overweight and dyslipidemia were for further exclusion. We speculated hs‐cTnT was a marker of the physiological renewal of cardiomyocytes.^38^ One recent study by Monneret et al^39^ also showed the value of hs‐cTnT was slightly higher in male, but in their Table 2, it was observed that women had higher concentrations than men in the elder people over 71 years of age group, which was the opposite of young people groups. Although the authors considered the difference as inexplicable because of the different age distribution, there were still some reasons to address. Firstly, as the author said age partitioning was clearly required, but the median of age of male was smaller than that of female (76 years old vs 80 years old, respectively). Secondly, analytical imprecision‐based approach might include some results in patients with stable coronary artery disease or other potentially stable heart disease. In this aspect, if the object of study was child or adolescents, potential diseases might not be factors when establishing 99th URLs of hs‐cTnT.^40^

Till now, there were no strong data to prove that the 99th percentile URL of hs‐cTnT by ethnicity should be established. These same or different conclusions whether caused by race or by comorbidities need further discussion. Gaggin et al^41^ concluded that there was little difference in hs‐cTnT between Americans and Vietnamese and previously deduced 99th percentile URL of hs‐cTnT could be used in Asian populations; however, the participants were recruited from self‐reported healthy volunteers or blood donors. Gore et al^36^ found African Americans had higher 99th percentile URL of hs‐cTnT than Caucasians, but patients with diabetes mellitus and hypertension were not excluded. If surrogate biomarkers were added, Gunsolus et al^42^ also found the difference between African American and Caucasian based on the AACC Universal Sample Bank; however, imaging, blood pressure, and lipid profile were not available. Due to lack of data support under the same selection criteria, it was hard for us to conclude that our Chinese‐based study could represent Asian value or be different from Americans and Europeans. It is well known that the more screening criteria used, the less 99th percentile URL of hs‐cTn would be.^6^, ^43^ If simply judged by the numbers of screening criteria, our Chinese study with more screening criteria had higher 99th percentile URL of hs‐cTnT than Koreans,^44^ Singaporeans,^43^ Americans, and Europeans.^4^, ^5^ It also indirectly illustrated the differences between Chinese and other races. But we thought unified selection criteria, which can avoid comorbidities, should be taken between races to judge whether ethnic‐specific 99th percentile URL of hs‐cTnT should be established. Together with AACC Universal Sample Bank, we also built sample bank based on improved selection criteria in the Chinese Reference Interval Study. We anticipated it would become a part of Universal Sample Bank to facilitate the comparison of hs‐cTn between manufacturers, healthcare providers, different countries, and races by eliminating variability among the normal populations selected for testing.^42^

This study has several limitations. Firstly, participants of male were 345 (37.0%) and participants more than 60 years old were less than other age group. But we still included more than 300 individuals of each gender, which met the requirements of 2018 AACC/IFCC criteria.^11^ For the age characteristics of the elderly, exclusion of comorbidities was a hard task. The stricter the screening criteria were, the fewer individuals would be included. We used stricter exclusion criteria than previous studies; in consequence, the number of elderly was rare. Secondly, we used FBG instead of HbA1c as the surrogate biomarker for diabetes. Our strategy was the same as Franzini et al^27^ that diabetes was defined as FBG more than 7 mmol/L or the use of any hypoglycemic agent. Thirdly except for ECG, we did not perform echocardiogram or MRI. Although ECG is definitely not a substitute for echocardiogram or MRI in diagnosis of CVD, we think ECG together with NT‐proBNP can somehow play an alternative role in determination of hs‐cTn 99th percentile URL.^28^, ^36^ Meanwhile, hs‐cTnT was associated with some diseases such as atrial fibrillation,^45^ which could be excluded by ECG not echocardiogram or MRI. Additionally, our research objective was to judge whether two 99th percentile URLs of hs‐cTnT established are different and both of them used the same standard, which might offset the influence of each other by choosing FBG and ECG.

In conclusion, compared with 2018 AACC/IFCC criteria, improved selection criteria through questionnaire survey, physical examination, and laboratory screening to further exclude hypertension, overweight and obesity, and dyslipidemia can avoid overestimation of the 99th percentile URL of hs‐cTnT. As to what extent it would affect clinical management and improve outcomes still needs further investigation.

## CONFLICT OF INTEREST

There are no conflicts of interest.
